# Enhancing Postpartum Mental Health: Evaluation of the Effect of Remote Peer Support Intervention

**DOI:** 10.1192/j.eurpsy.2024.566

**Published:** 2024-08-27

**Authors:** H. Němcová, K. Hrdličková, M. Kuklová, A. Horáková, E. Nosková, P. Švancer, N. Byatt, A. Šebela

**Affiliations:** ^1^National Institute of Mental Health, Klecany; ^2^Faculty of Arts, Department of Psychology; ^3^Faculty of Science, Department of Demography and Geodemography; ^4^Second Faculty of Medicine; ^5^First Faculty of Medicine; ^6^Third Faculty of Medicine, Charles University, Prague, Czech Republic; ^7^Department of Psychiatry, UMass Chan Medical School and UMass Memorial Health, Worcester, United States

## Abstract

**Introduction:**

The postpartum period poses a risk of both onset and relapse of mental health disorders in mothers, which can impact maternal-child relationships and development of children. Timely intervention is crucial, especially considering that majority of at-risk women do not seek professional help.

**Objectives:**

This study aims to evaluate the effectiveness of Mom Supports Mom, a remote peer support intervention, in improving the mental health of postpartum women.

**Methods:**

A randomized controlled trial with 488 Czech postpartum women with depressive symptoms (Edinburgh Postnatal Depression Scale, EPDS score ≥ 10 shortly after giving birth) assessed the impact of Mom Supports Mom on depressive and anxiety symptoms (EPDS and Perinatal Anxiety Screening Scale, PASS) and health-related quality of life (Assessment of Quality of Life, AQoL-8D) at 6 weeks postpartum. The Mini-International Neuropsychiatric Interview 5 (MINI) was used to assess psychiatric diagnoses.

**Results:**

The intervention significantly reduced depressive (Cohen’s d = 0.30; p = 0.003) and anxiety symptoms (Cohen’s d = 0.29; p = 0.003) and improved health-related quality of life (Cohen’s d = 0.27; p = 0.008) at 6 weeks postpartum. No significant difference was observed in psychiatric diagnoses between the intervention and the control group.

**Conclusions:**

Mom Supports Mom intervention reduces postpartum depressive and anxiety symptoms and enhances health-related quality of life. These findings support the integration of peer support into perinatal mental health care, addressing barriers that women face in seeking help.

**Disclosure of Interest:**

None Declared

